# Right Ventricular and Right Atrial Strain Are Associated with Kidney Dysfunction in Acute Heart Failure

**DOI:** 10.3390/diagnostics14141576

**Published:** 2024-07-21

**Authors:** Vasileios Anastasiou, Emmanouela Peteinidou, Christos Tountas, Stylianos Daios, Dimitrios V. Moysidis, Emmanouil Fardoulis, Christos Gogos, Marieta Theodorakopoulou, Fotini Iatridi, Pantelis Sarafidis, George Giannakoulas, Theodoros Karamitsos, Victoria Delgado, Antonios Ziakas, Vasileios Kamperidis

**Affiliations:** 1 1st Department of Cardiology, School of Medicine, Faculty of Health Sciences, Aristotle University of Thessaloniki, AHEPA Hospital, St. Kiriakidi 1, 546 36 Thessaloniki, Greece; vasianas44@gmail.com (V.A.); emma2405@windowslive.com (E.P.); stylianoschrys.daios@gmail.com (S.D.); mf.pred@hotmail.com (E.F.); cgogos.cardio@gmail.com (C.G.); ggiannakoulas@gmail.com (G.G.); karamits@gmail.com (T.K.); tonyziakas@hotmail.com (A.Z.); 2 Cardiology Department, Sismanoglio Hospital, 151 26 Athens, Greece; tountasxristos@yahoo.gr; 3 424 General Military Hospital, 564 29 Thessaloniki, Greece; dimoysidis@gmail.com; 4 Department of Nephrology, School of Medicine, Aristotle University of Thessaloniki, Hippokration Hospital, 546 42 Thessaloniki, Greece; marietatheod@gmail.com (M.T.); fotini.iatridi@gmail.com (F.I.); psarafidis11@yahoo.gr (P.S.); 5 Department of Cardiology, Hospital University Germans Triasi Pujol, 08916 Barcelona, Spain; vdelgadog.germanstrias@gencat.cat

**Keywords:** acute heart failure, renal impairment, increased renal afterload, right ventricular strain, right atrial strain

## Abstract

Background: In acute heart failure (HF), low cardiac output and venous congestion are pathophysiological mechanisms that contribute to renal function impairment. This study investigated the association between advanced echocardiographic measures of right ventricular and atrial function and renal impairment in patients with acute HF. Methods and Results: A total of 377 patients hospitalized for acute HF were prospectively evaluated. Estimated glomerular filtration rate (eGFR) on admission was measured using the 2021 Chronic Kidney Disease Epidemiology Collaboration creatinine equation. Advanced echocardiographic assessment was performed on admission. Patients with eGFR < 45 mL/min/1.73 m^2^ were more likely to have chronic heart failure, chronic atrial fibrillation, and type 2 diabetes mellitus compared to patients with eGFR ≥ 45 mL/min/1.73 m^2^. Patients with lower eGFR had lower cardiac output, higher mean E/e’ ratio, larger right ventricular (RV) size, worse RV free wall longitudinal strain, more impaired right atrial (RA) reservoir strain, and more frequent severe tricuspid regurgitation. RV free wall longitudinal strain and RA reservoir strain were the only independent echocardiographic associates of low eGFR, whereas cardiac output was not. Conclusions: Impaired RV and RA longitudinal strain were independently associated with eGFR < 45 mL/min/1.73 m^2^ in acute HF, while reduced cardiac output was not. This suggests that RV and RA dysfunction underlying venous congestion and increased renal afterload are more important pathophysiological determinants of renal impairment in acute HF than reduced cardiac output.

## 1. Introduction

Patients with acute heart failure (HF) are an increasing group with a remarkably high rate of in-hospital mortality [1], especially when associated with renal impairment on admission [2,3]. The pathophysiological mechanism of renal dysfunction in acute HF is complex and remains poorly defined. Impaired kidney perfusion, arterial hypovolemia, and reduced afferent arteriolar flow have a detrimental effect on the estimated glomerular filtration rate (eGFR) [4]. However, more recent data have challenged this hypothesis, indicating that the increased renalvenous pressureand the venous congestionare the determinants of renal impairment [5].

Echocardiography is a universally available tool that can be easily utilized to derive information on hemodynamics and venous congestion. Beyond the assessment of left ventricular (LV) function, cardiac output can be non-invasively calculated and is closely related with end-organ perfusion [6]. Additionally, echocardiography can thoroughly assess right ventricular (RV) function, which is tightly linked with splanchnic congestion [7]. Previous evidence has shown a strong correlation between eGFR and RV function, as assessed by tricuspid annular plane systolic excursion [8]. However, strain imaging of the RV has recently demonstrated a superior correlation with renal function compared to standard RV function indices [9]. Moreover, strain imaging can be used to evaluate RA function, which has been independently associated with venous congestion [10]. As recently demonstrated, impaired RA function, assessed by RA strain, was independently associated with abnormal hepatic and renal vein flow patterns, indicating excess venous congestion [11].

Whether reduced renal preload due to impaired LV function and cardiac output or augmented renal afterload due to RV and RA dysfunction predominantly affects the estimated eGFR in acute HF is yet to be defined. Therefore, this study sought to investigate if advanced echocardiography with strain imaging could provide further insights into the pathophysiology of renal impairment in the setting of acute HF.

## 2. Methods

### 2.1. Study Population

Consecutive acute HF subjects hospitalized at the cardiology department of AHEPA University General Hospital in Thessaloniki, Greece, from 04/2022 until 11/2023 were enrolled if they fulfilled the prespecified inclusion criteria, which have been previously outlined (‘’Beyond-Myo HF Study’’—https://www.clinicaltrials.gov/study/NCT05573997, URL accessed on 10 October 2022) [12]. The present study adheres to the principles of the Declaration of Helsinki (2013 Amendment). The conduction of this study was formally assessed and authorized by the Ethics Committee of the School of Medicine of Aristotle University of Thessaloniki (Approval number: 19/2022). Patients were informed and formally consented to take part in this study.

Clinical characteristics, laboratory indices, and the therapeutic management of the patients were recorded. The 2021 Chronic Kidney Disease Epidemiology Collaboration (CKD-EPI) creatinine equation [13] was used to assess the estimatedeGFR of the study sample as recommended by the National Kidney Foundation and the American Society of Nephrology [14]. The first available blood sample on admission was utilized for the estimation of eGFR.

### 2.2. Echocardiographic Analysis

An advanced echocargiographic assessment was prospectively conducted in all participants on the first day of admission using Vivid E95 or Vivid S70 ultrasound systems (GE Healthcare, Chicago, IL, USA). The echocardiographic data were analyzed offline using an EchoPac112.0.1 workstation (GE Medical Systems, Horten, Norway). All analyses were undertaken by a cardiologist with expertise in cardiovascular imaging, who was blinded to the laboratory and clinical characteristics of the participants.

Updated recommendations were followed to acquire and analyze all relevant echocardiographic data, including dimensions and Doppler data [15,16,17]. In particular, Simpson’s biplane method was used to estimate the LV ejection fraction. The biplane method was employed to measure left atrial maximum volume at an end-systolic apical four- and two-chamber frame [15]. Stroke volume was derived from left ventricular outflow tract diameter and velocity time integral, and by multiplying for heart rate, thecardiac output was derived. The relevant guidelines for the assessment of valvular heart disease were used to estimate left-sided valvular disease severity [17].

An RV-focused view was used to measure metrics of the RV size and function. All recommended metrics of RV function were calculated, including fractional area change, tricuspid annular peak systolic excursion, and systolic movement of the RV free wall by tissue Doppler imaging (S’) [18]. For the fractional area change frames for the RV, end-diastole and end-systole were used [18]. M-mode and tissue Doppler imaging were employed to calculate tricuspid annular peak systolic excursionand S’ [18]. Dimensions of the RV were measured at end-diastole frame at three levels tricuspid annulus, RV base, and RV mid-diameter [18]. Transtricuspid measurements of the RV E wave were taken using pulse-wave Doppler [18]. Right atrial (RA) volume was evaluatedat an end-systolic frame [18]. Grading of tricuspid regurgitation severity was performed as suggested in updated recommendations [16,17]. The simplified Bernoulli equation was used for pulmonary artery systolic pressure assessment, and the diameter and inspiratory variationof the inferior vena cava were assessed to evaluate the RA pressure [18].

Speckle-tracking data are reported in absolute numbers (i.e., positive). LV global longitudinal strain was measured using four-, three-, and two-chamber views [19]. A 16segment model was used by the software to divide the myocardium, and their average number was reported as LV global longitudinal strain.

RV global longitudinal strain values were calculated from the basal, mid, and apical segments of the RV free wall and the septum [20]. For the RV free wall longitudinal strain, the average of the 3 free-wall segments was used, and septal segments were excluded [20].

Left atrial strain analysis was undertaken using loops from the apical four-chamber and apical two-chamber view, and subsequently, a six-segment model was automatically generated. Peak left atrial strain values were calculated by averaging these 6 segments. In a similar manner, for the RA strain, the RA was traced in an RV-focused view, and the left atrial strain software was applied. Given the high prevalence of atrial fibrillation in this study, values for reservoir strain were assessed and presented.

### 2.3. Statistical Analysis

The sample size was divided into 2 cohorts according to their estimated eGFR. A cut-off eGFR value of 45 mL/min/1.73 m^2^ was used to dichotomize the population. Categorical variables are reported as frequencies (percentages) and were compared with the chi-squared test. Continuous variables are presented as mean ± standard deviation when they follow normal distribution and as median (interquartile range) when they follow a non-normal distribution. Normality of distribution was assessed for all variables. The unpaired Student *t*-test and the Mann–Whitney U test were employed to compare continuous variables between the 2 groups. Pearson’s chi-squared test was used in case of categorical variables.

To identify associates of eGFR < 45 mL/min/1.73 m^2^, univariable logistic regression analysis was applied using clinically relevant baseline and echocardiographic characteristics. Variables that demonstrated statistical significance in the univariable analysis were entered in a multivariable model to identify independent predictors of renal impairment in acute HF. The odds ratio, including the 95% confidence interval, and the *p*-value are presented in the tables.

All tests were two-sided, and the threshold of *p* < 0.05 was used to define statistical significance. SPSS software, version 25 (IBM SPSS Statistics, for Windows, Armonk, NY, USA), and R version 3.4.4 (R Foundation for Statistical Computing, Vienna, Austria) were employed for the statistical analyses.

## 3. Results

### 3.1. Clinical and Echocardiographic Characteristics

Overall, 377 consecutive hospitalized patients with acute HF were prospectively assessed (mean age 73.6 ± 11.9, 60.5% male). Of these, 252 patients had eGFR ≥ 45 mL/min/1.73 m^2^, whereas 125 patients had eGFR < 45 mL/min/m^2^ on admission. Table 1 compares the baseline characteristics of the two cohorts. Patients with eGFR < 45 mL/min/1.73 m^2^ were older, had lower body surface area, and were more likely to have higher New York Heart Association class symptoms. Regarding their past medical history, the cohort with eGFR < 45 mL/min/1.73 m^2^ had more frequent chronic atrial fibrillation, chronic heart failure, and type 2 diabetes mellitus, while they presented less frequently with new-onset heart failure. Their medication more frequently comprised furosemide with higher doses and mineralocorticoid receptor antagonists. Based on their laboratory findings on admission, patients with eGFR < 45 mL/min/1.73 m^2^ had higher levels of NT-pro-BNP, troponin I, creatitine, blood urea nitrogen, and serum potassium, as well aslower hemoglobin levels.

**Figure 1:**
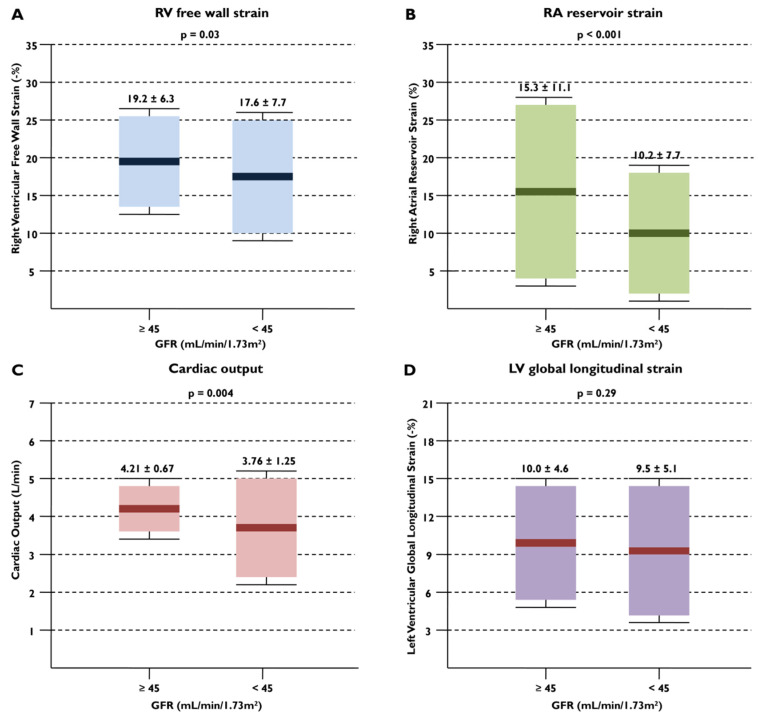
Patients with glomerular filtration rate (eGFR) < 45 mL/min/1.73 m^2^ had significantly more impaired indices of right ventricular (RV) (**A**) and right atrial (RA) (**B**) strain, as well ascardiac output (**C**), compared to patients with eGFR ≥ 45 mL/min/1.73 m^2^. No differences were shown in left ventricular (LV) global longitudinal strain between the 2 groups (**D**).

**Figure 2:**
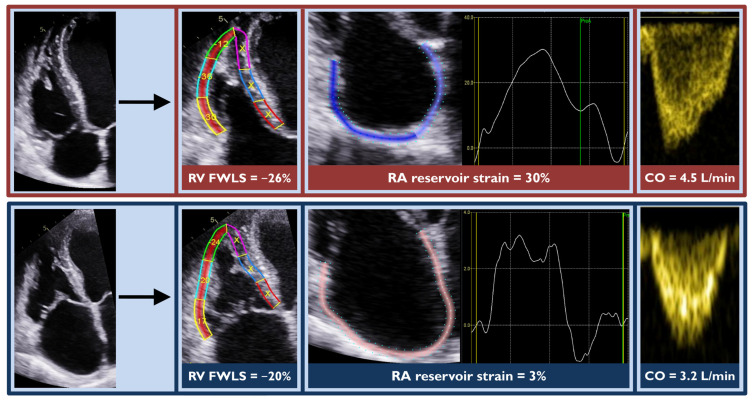
An example highlighting the main echocardiographic differences between a patient with glomerular filtration rate (eGFR) ≥ 45 mL/min/1.73 m^2^ (top row) and a patient with eGFR < 45 mL/min/1.73 m^2^ (bottom row) with acute heart failure is demonstrated. The patient with eGFR < 45 mL/min/1.73 m^2^ demonstrated more impaired right ventricular (RV) free wall strain, more impaired right atrial (RA) reservoir strain, and lower cardiac output (CO).

Table 2 describes the baseline echocardiographic characteristics of the study groups. Patients with eGFR < 45 mL/min/1.73 m^2^ had significantly lower cardiac output and higher mean E/e’, indicating higher filling pressures compared to patients with eGFR ≥ 45 mL/min/1.73 m^2^. Notably, none of the LV size and function parameters significantly differed between the two cohorts. In terms of RV size, patients with eGFR < 45 mL/min/1.73 m^2^ had significantly larger RV end-diastolic and end-systolic areas. Likewise, their basal and mid RV end-diastolic diameter was larger. Most indices of RV function such as fractional area change, tricuspid annular plane systolic excursion, and RV free wall longitudinal strain were significantly more impaired for patients with eGFR < 45 mL/min/1.73 m^2^ compared to their counterparts (Table 2). Pulmonary valve acceleration time was shorter for patients with the worst eGFR. Patients with eGFR < 45 mL/min/1.73 m^2^ demonstrated significantly more impaired RA reservoir strain, whereas none of the other atrial parameters differed between the twogroups. Overall, there were significant differences in the RV and RA strain parameters, as well ascardiac output, between the two groups, with no difference in the LV strain (Figure 1). Additionally, patients in the low-eGFR group were more likely to have severe tricuspid regurgitation. An example demonstrating the main echocardiographic differences between a patient with low and a patient with high eGFR is shown in Figure 2.

### 3.2. Independent Associates of Low eGFR

Table 3 summarizes the baseline clinical and echocardiographic variables that demonstrated independent association with eGFR < 45 mL/min/1.73 m^2^. Clinical, laboratory, and echocardiographic parameters were entered in the univariable linear regression analysis. Only variables that indicated association with eGFR < 45 mL/min/1.73 m^2^ were entered in the multivariable analysis. Regarding clinical parameters, chronic heart failure, type 2 diabetes mellitus, and NT-pro-BNP retained independent association with low eGFR. Of note, only metrics of RV function including RV free wall longitudinal strain and RA reservoir strain emerged as independent echocardiographic associates of low eGFR, whereas metrics of LV function such as cardiac output lost their association after adjustment.

## 4. Discussion

This prospective study demonstrated that patients admitted to the hospital due to acute HF with eGFR < 45 mL/min/1.73 m^2^ presented with more impaired measures of RV size and function compared to their counterparts, whereas measures of LV function did not differ across the groups of eGFR. On admission for acute HF, RV free wall longitudinal strain and RA reservoir strain were independent echocardiographic associates of eGFR < 45 mL/min/1.73 m^2^ after adjusting for clinical and biochemical variables, whereas cardiac output and LV function was not.

### 4.1. Cardiac Output and Renal Impairment

The strong association of renal impairment with poor outcomes in acute HF is well known [21]. Impaired renal preload is the most widely postulated mechanism affecting eGFR in HF. Reduced cardiac output secondary to LV systolic function is associated with inadequate afferent arteriolar flow, which causes direct ischemic insult of the glomerulus and impairs eGFR [4]. In the current analysis, patients in the low-eGFR group demonstrated worse cardiac output compared to patients in the high-eGFR group.

While cardiac output was associated with eGFR < 45 mL/min/1.73 m^2^ in the univariable model, the statistical significance was lost when adjusted for clinical variables and measures of RV function. This observation challenges the hypothesis of renal hypoperfusion as the primary mechanism of renal dysfunction in acute HF. However, even in this scenario, RV dysfunction leads to impaired forward pulmonary flow and as a consequence to low cardiac output, arterial renal hypoperfusion, and low eGFR. Thus, the low cardiac output could be the sequelae of RV and not merely LV dysfunction [22,23].

### 4.2. Venous Congestion and Renal Impairment

Beyond kidney hypoperfusion, the alternative hypothesis of congestive renal impairment in acute HF has gained attention and has been supported by invasive hemodynamic evidence [5,24]. Historically, the idea was that elevated central venous pressure can be backward transmitted to therenal veins and lead to direct impairment of renal function [25]. As has been recently shown, splachnic congestion may lead to abnormalities of hepatic and renal vein flow that could adversely impact organ function if left untreated [11]. This notion was corroborated by Mullens et al., who demonstrated in a cohort of patients with low-output decompensated acute HF that increased central venous pressure on admission was the strongest associated factor of worsening renal function, while invasively derived cardiac index had a limited impact on renal function [5]. Further evidence from a large cohort of over 2500 patients undergoing right heart catheterization underlined the independent association of central venous pressure with estimated eGFR, indicating increased renal afterload as the core etiological pathway to renal impairment in HF [24].

The current analysis used advanced echocardiography to underpin the argument of congestive renal impairment in acute HF by highlighting the independent association of RV and RA function with low eGFR. The RV longitudinal strain has been recognized as a sensitive marker of RV function and a strong outcome predictor in HF [26]. As was shown, the impaired RV free wall longitudinal strain and impaired RA reservoir strain were independently associated with eGFR < 45 mL/min/1.73 m^2^ along with clinical factors including chronic heart failure, type 2 diabetes mellitus, and increased NT-pro-BNP (Table 3). RV dysfunction and elevated RA pressure have previously been linked with splanchnic congestion, connoting an advanced stage of HF [7]. Adverse RV and RA remodeling have been shown to cause abnormalities of the hepatic and renal vein flow patterns, which connote splachnic congestion [11]. In particular, they can cause systolic wave reversal of the hepatic veins, increased pulsatility fraction of the portal vein Doppler flow, and discontinuous interlobar renal vein Doppler flow pattern, all indicative of excess congestion [11]. The present analysis adds to these previous findings, suggesting that renal impairment in the setting of acute HF is usually a sequela of advanced congestion and predominantly a sign of disease chronicity expressed as RV and RA dysfunction. On the other hand, LV dysfunction and reduced cardiac output seem to be outweighed by RV and RA dysfunction and play a secondary role in the pathophysiology of renal impairment in acute HF, which hasto be further studied.

### 4.3. Clinical Implications

The current study highlights the prominent role of RV and RA function in the development of cardiorenal syndrome in acute HF. These findings may help explain why practices of efficient and safe fluid removal in acute HF including loop diuretics or ultrafiltraton are linked with eGFR improvements, whereas underdiuresis and persistently increased central venous pressure may adversely affect eGFR [27]. In this regard, future research should focus on practices that improve RV function and effectively alleviate augmented renal afterload to improve outcomes after admission for acute HF.

### 4.4. Limitations

Although a prospective study, it is single-center study, and its results require confirmation and external validation from other researchers. Additionally, eGFR was merely estimated using the blood sample on admission for acute HF and was associated with echocardiographic data acquired within 24 h from admission. However, this strategy allowed the capturing of patients at a state of maximum congestion before the introduction of acute HF medication.

## 5. Conclusions

In a cohort of hospitalized patients with acute HF, RV free wall longitudinal strain and RA reservoir strain were independently associated with renal impairment on admission, whereas indices of LV function including cardiac output and LV longitudinal strain were not. RV and RA dysfunction, leading primarily to venous congestion and increased renal afterload, may be the main driving force of renal impairment in acute HF, compared to LV dysfunction, reduced cardiac output, and reduced renal preload.

## Figures and Tables

**Table 1:** Baseline clinical characteristics based on estimated glomerular filtration rate (eGFR) at admission.

Variable	All (*n* = 377)	eGFR ≥ 45 mL/min/1.73 m^2^ (*n* = 252)	eGFR < 45 mL/min/1.73 m^2^ (*n* = 125)	*p*-Value
Clinical Characteristics				
Age, years	73.6 ± 11.9	71.4 ± 12.5	77.8 ± 9.4	<0.001
Male, n (%)	228 (60.5)	159 (63.1)	69 (55.2)	0.15
BSA, m^2^	1.93 ± 0.23	1.94 ± 0.23	1.90 ± 0.23	0.048
Systolic blood pressure, mmHg	124.1 ± 19.0	124.4 ± 18.1	123.0 ± 19.8	0.51
Diastolic blood pressure, mmHg	74.0 ± 15.5	75.6 ± 16.0	70.6 ± 14.1	0.004
Heart rate, bpm	82.6 ± 18.0	83.9 ±18.0	80.3 ± 17.9	0.07
NYHA III-IV, %	129 (34.2)	70 (27.8)	59 (47.2)	<0.001
Past Medical History				
Ischemic heart disease, n (%)	145 (38.4)	97 (38.5)	48 (38.4)	1.00
Dilated cardiomyopathy, n (%)	41 (10.9)	27 (10.7)	14 (11.2)	0.86
New-onset heart failure, n (%)	164 (43.5)	133 (52.8)	31 (24.8)	<0.001
Chronic heart failure, n (%)	211 (56.0)	118 (46.8)	93 (76.8)	<0.001
Severe valvular heart disease, n (%)	77 (20.4)	52 (20.6)	25 (20.0)	1.00
Chronic atrial fibrillation, n (%)	98 (26.0)	54 (21.4)	44 (35.2)	0.006
Hypertension, n (%)	196 (52.0)	124 (49.2)	72 (57.6)	0.13
Type 2 diabetes mellitus, n (%)	136 (36.1)	73 (29.0)	63 (50.4)	<0.001
Chronic obstructive pulmonary disease, n (%)	58 (15.4)	40 (15.9)	18 (14.4)	0.76
Medication on Admission				
Furosemide, n (%)	168 (44.6)	100 (39.7)	68 (54.4)	0.019
Furosemide dose, mg/day (range)	38 (0–40)	32 (0–40)	53 (0–80)	0.002
ACEi/ARBs, n (%)	126 (33.4)	83 (32.9)	43 (34.4)	0.82
ARNI, n (%)	32 (8.5)	17 (6.7)	15 (12.0)	0.12
MRAs, n (%)	102 (27.1)	57 (22.6)	45 (36.0)	0.007
B-blockers, n (%)	164 (43.5)	103 (40.9)	61 (48.8)	0.15
SGLT2i, n (%)	76 (20.2)	43 (17.1)	33 (26.4)	0.08
Laboratory Indices				
NT-pro-BNP, pg/mL (range)	4376 (2113–10,697)	3199 (1701–7064)	9387 (3985–25,246)	<0.001
Admission troponin I, ng/L (range)	40 (25–81)	36 (22–69)	55 (36–97)	<0.001
AST, U/L (range)	27 (20–39)	28 (20–38)	26 (20–40)	0.63
ALT, U/L (range)	20 (13–33)	21 (14–35)	17 (11–30)	0.06
CPK, U/L (range)	76 (46–149)	83 (48–150)	71 (45–142)	0.19
Creatitine, mg/dL	1.4 ± 0.65	1.10 ± 0.24	2.01 ± 0.79	<0.001
Blood urea nitrogen, mg/dL (range)	56 (40–79)	47 (37–61)	94 (62–122)	<0.001
Sodium, mEq/L	136.8 ± 12.6	137.3 ± 12.5	135.7 ± 12.9	0.25
Potassium, mEq/L	4.3 ± 0.6	4.2 ± 0.6	4.4 ± 0.7	0.011
Hemoglobin, g/dL	12.3 ± 2.2	12.6 ± 2.1	11.6 ± 2.1	<0.001
Platelets, K/dL (range)	222 (174–281)	229 (182–285)	209 (166–266)	0.06

Continuous variables are reported as median (IQR) or mean ± SD and categorical variables as percentages. Abbreviations: ACEi, angiotensin-converting enzyme inhibitors; ALT, alanine aminotransferase; ARBs, angiotensin receptor blockers; ARNI, angiotensin receptor/neprilysininhibitor; AST, aspartate aminotransferase; BSA, body surface area; CPK, creatine phosphokinase; eGFR, glomerular filtration rate; MRAs, mineralocorticoid receptor antagonists; NT-pro-BNP, N-terminal pro-brain natriuretic peptide; NYHA, New York Heart Association; SGLT2i, sodium-glucose co-transporter-2inhibitors.

**Table 2:** Baseline echocardiographic characteristics based on estimated glomerular filtration rate (eGFR) atadmission.

Variable	All (n = 377)	eGFR ≥ 45 mL/min/1.73 m^2^ (n = 252)	eGFR < 45 mL/min/1.73 m^2^ (n = 125)	*p*-Value
LV end-diastolic diameter, mm	55.7 ± 11.5	55.6 ± 11.5	55.9 ± 11.7	0.80
LV end-systolic diameter, mm	45.1 ±12.6	45.2 ± 12.3	45.2 ± 13.3	1.00
LV end-diastolic volume indexed, mL/m^2^	87.9 ± 37.8	86.8 ± 37.4	90.6 ± 39.2	0.36
LV end-systolic volume indexed, mL/m^2^	57.3 ± 35.3	56.4 ± 34.7	59.5 ± 37.0	0.42
LV ejection fraction, %	39.4 ± 14.7	39.6 ± 14.1	39.1 ± 15.7	0.75
LV GLS, %	9.9 ± 4.8	10.0 ± 4.6	9.5 ± 5.1	0.29
Stroke volume, mL	50.37 ± 18.44	51.46 ± 18.64	48.26 ± 18.19	0.12
Cardiac output, L/min	4.06 ±1.40	4.21 ± 0.67	3.76 ± 1.25	0.004
E, cm/s	102.6 ± 27.1	101.7 ± 27.6	104.6 ± 26.3	0.34
A, cm/s	58.7 ± 28.0	57.2 ± 26.1	62.3 ± 31.7	0.18
E/A	2.06 ±1.15	2.08 ± 1.19	1.99 ± 1.06	0.60
Mean E/e’	18.9 ± 6.5	18.2 ± 6.0	20.5 ± 7.3	0.002
RV end-diastolic area, cm^2^	22.8 ±7.1	22.2 ± 6.9	24.0 ± 7.4	0.021
RV end-systolic area, cm^2^	15.4 ± 6.1	14.8 ± 5.9	16.5 ± 6.5	0.016
Tricuspid annular diameter, mm	35.5 ± 7.5	34.9 ±7.5	36.7 ±7.4	0.042
Basal RV end-diastolic diameter, mm	46.3 ± 8.1	45.5 ± 8.2	47.8 ± 7.8	0.01
Mid RV end-diastolic diameter, mm	34.7 ± 8.7	34.1 ± 8.7	36.0 ± 8.8	0.05
Apex-to-base RV end-diastolic diameter, mm	76.4 ± 13.5	76.7 ± 13.5	75.9 ±13.8	0.62
Fractional area change, %	33.7 ± 9.6	34.3 ± 9.3	32.7 ± 10.0	0.14
S’TDI tricuspid, cm/s	9.5 ± 3.0	9.8 ± 2.8	8.8 ± 3.1	0.002
TAPSE, mm	16.5 ± 4.1	16.9 ± 3.9	15.8 ± 4.5	0.015
RV E, cm/s	57.9 ± 18.9	56.6 ± 17.7	60.4 ± 20.4	0.10
PV acceleration time, ms	83.9 ± 24.1	86.7 ± 24.8	78.3 ± 22.2	0.002
RV free wall LS, %	18.6 ± 6.8	19.2 ± 6.3	17.6 ± 7.7	0.03
RV GLS, %	14.8 ± 5.4	15.1 ± 5.1	14.1 ± 5.9	0.08
LA end-systolic volume indexed, mL/m^2^	58.2 ± 20.7	56.8 ± 20.1	61.2 ± 22.1	0.06
LASr, %	8.5 ± 4.5	8.9 ± 4.5	8.0 ± 4.5	0.07
RA volume, mL	86.4 ± 49.6	84.2 ± 50.8	90.4 ± 47.4	0.26
RASr, %	13.6 ± 10.4	15.3 ± 11.1	10.2 ± 7.7	<0.001
Severe left-sided heart disease, %	77 (20.4)	44 (17.4)	30 (24.0)	0.08
Severe tricuspid regurgitation, %	146 (38.7)	86 (34.0)	60 (48.0)	0.005
RV systolic pressure, mmHg	49.3 ± 14.0	48.4 ± 14.0	51.1 ± 13.9	0.08
Inferior vena cava diameter, mm	23.1 ± 5.8	22.7 ± 5.6	23.9 ± 6.2	0.06

Continuous variables are reported as median (IQR) or mean ± SD and categorical variables as percentages. Abbreviations: eGFR, glomerular filtration rate; GLS, global longitudinal strain; LASr, left atrial reservoir strain; LS, longitudinal strain; LV, left ventricular; PV, pulmonary valve; RASr, right atrial reservoir strain; RV, right ventricular; TAPSE, tricuspid annular plane systolic excursion; TDI, tissue Doppler imaging.

**Table 3:** Univariable and multivariable logistic regression for associates of eGFR < 45 mL/min/1.73 m^2^.

	Univariable			Multivariable		
Variable	OR	95% CI	*p*-Value	OR	95% CI	*p*-Value
Systolic blood pressure, mmHg	1.00	0.99–1.02	0.51			
Diastolic blood pressure, mmHg	1.02	1.01–1.04	0.005	1.02	0.99–1.04	0.14
NHYA III, IV	2.32	1.49–3.63	<0.001	1.43	0.76–2.70	0.27
Chronic heart failure	3.30	2.06–5.29	<0.001	2.36	1.15–4.89	0.019
Chronic atrial fibrillation	1.99	1.24–3.20	0.004	1.03	0.51–2.10	0.93
Type 2 diabetes mellitus	2.49	1.60–3.88	<0.001	2.52	1.38–4.62	0.003
NT-pro-BNP, pg/mL *	5.65	3.27–9.78	<0.001	5.78	2.84–11.63	<0.001
LV end-diastolic volume indexed, mL/m^2^	1.00	0.99–1.01	0.36			
LV ejection fraction, %	0.99	0.98–1.01	0.75			
LV GLS, %	0.98	0.93–1.02	0.29			
Stroke volume, mL	1.01	0.99–1.02	0.12			
Cardiac output, L/min	0.79	0.67–0.93	0.005	0.95	0.75–1.20	0.65
Mean E/e’ ratio	1.06	1.02–1.09	0.003	1.01	0.96–1.06	0.78
Basal RV end-diastolic diameter, mm	1.04	1.01–1.06	0.011	1.04	0.99–1.07	0.12
Fractional area change, % **	0.98	0.96–1.01	0.14			
S’TDI tricuspid, cm/s **	0.88	0.82–0.96	0.002			
TAPSE, mm **	0.94	0.89–0.99	0.016			
RV free wall LS, %, % **	0.97	0.93–0.99	0.031	0.94	0.89–0.99	0.027
RV GLS, % **	0.96	0.93–1.00	0.08			
LA volume indexed, mL/m^2^	1.01	1.00–1.02	0.06			
LASr, %	0.95	0.91–1.00	0.07			
RA volume, mL	1.00	0.99–1.01	0.26			
RASr, %	0.95	0.92–0.97	<0.001	0.95	0.91–0.99	0.029
Severe left heart valvular disease	1.51	0.89–2.55	0.12			
Severe tricuspid regurgitation	1.82	1.17–2.82	0.008	2.10	0.96–4.57	0.06
RV systolic pressure, mmHg	1.01	0.99–1.03	0.08			

Age was not included in the logistic regression analysis, as it is part of the eGFR equation. * The logarithmic transform of NT-pro-BNP was used. ** Only RV free wall longitudinal strain was used between RV function variables to be incorporated in the multivariable analysis as a novel, sensitive index of RV systolic function. Abbreviations: eGFR, glomerular filtration rate; GLS, global longitudinal strain; LV, left ventricular; LA, left atrium; LASr, left atrial strain reservoir; LS, longitudinal strain; NT-pro-BNP, N-terminal pro-brain natriuretic peptide; NYHA, New York Heart Association; RA, right atrium; RASr, right atrial strain reservoir; RV, right ventricular; TASPE, tricuspid annular plane systolic excursion; TDI, tissue Doppler imaging.

## Data Availability

The deidentified participant data will be shared on a request basis. Please directly contact the corresponding author to request data sharing.

## Abbreviations

CKD-EPI	Chronic Kidney Disease Epidemiology Collaboration
eGFR	estimated glomerular filtration rate
HF	heart failure
LV	left ventricular/ventricle
NT-pro-BNP	N-terminal pro B-type natriuretic peptide
RA	right atrial/atrium
RV	right ventricular/ventricle
